# Heparin-Independent and Heparin-Dependent Anti-CXCL4 Antibodies Have a Reciprocal Expression in a Systemic Sclerosis Patients’ Cohort

**DOI:** 10.3390/antib11040077

**Published:** 2022-12-15

**Authors:** Raffaella Palazzo, Katia Stefanantoni, Marius Cadar, Alessia Butera, Valeria Riccieri, Roberto Lande, Loredana Frasca

**Affiliations:** 1 Istituto Superiore di Sanità, National Center for Drug Research and Evaluation, Viale Regina Elena, 299, 00161 Rome, Italy; 2 Department of Clinical, Internal, Anesthesiological and Cardiovascular Sciences, Sapienza University of Rome, Viale del Policlinico, 155, 00161 Rome, Italy

**Keywords:** SSc, heparin-dependent antibodies, heparin-independent antibodies

## Abstract

Systemic sclerosis (SSc) is a chronic disease characterized by skin/internal organ fibrosis, vasculopathy and autoimmunity. Chemokine (C-X-C motif) ligand 4 (CXCL4) is an early SSc biomarker that predicts worse disease outcome. We previously reported that CXCL4 is an autoantigen in SSc, and anti-CXCL4 antibodies correlated with IFN-I and were more abundant in patients with lung fibrosis. However, it is unclear whether antibodies to CXCL4 in SSc are only directed to CXCL4 or recognize complexes formed by CXCL4 and heparin. Here, by analyzing an SSc cohort, we addressed the occurrence of circulating heparin-dependent VS heparin-independent anti-CXCL4 antibodies and their relationship with a few disease parameters. We found that heparin-dependent, like the heparin-independent antibodies, are higher in SSc as compared to healthy donors; they are detectable in 24% and 30% of the SSc patients, respectively, and appear inversely correlated and mutually exclusive. Like the heparin-independent antibodies, heparin-dependent antibodies correlated with digital ulcers. However, in contrast to heparin-independent antibodies, heparin-dependent antibodies did not correlate with IFN-I, but were largely expressed in patients with pulmonary arterial hypertension. This pilot study indicates that heparin-dependent antibodies are worth studying in larger SSc cohorts to address whether they discriminate SSc sub-groups with different pathological characteristics and outcomes.

## 1. Introduction

Systemic sclerosis (SSc) is a chronic disease characterized by skin and internal organ fibrosis, vasculopathy and autoimmunity [1,2]. Chemokine (C-X-C motif) ligand 4 (CXCL4) is an early SSc biomarker, which predicts a worse disease prognosis and later complications, such as pulmonary fibrosis [3,4,5]. CXCL4 binds DNA and RNA and favors interferon (IFN)-α production by plasmacytoid dendritic cells (pDC), contributing to the type I IFN (IFN-I) signature present in about half of the SSc patients [5,6]. Importantly, it has been shown that presence of an IFN-I signature early in the disease is often associated with more aggressive disease at later stages and complications [3,7,8,9,10]. Thus, both CXCL4 and IFN-I seem to play a role in early stages and be associated with future complications. Importantly, CXCL4 and IFN-I are closely linked via the process of triggering the nucleic sensor TLRs that leads to the IFN-I signature. In addition to that, we have previously shown that CXCL4 also acts as B-cell autoantigen in SSc patients [11,12]. This could be the result of formation of large CXCL4-DNA complexes in SSc patients, which could contribute to the immunogenicity of CXCL4. Indeed, we have previously shown that CXCL4-DNA complexes circulate in SSc patients, correlate with IFN-I and are also present in SSc-affected skin where they co-localize with an IFN-I induced gene [5]. Anti-CXCL4 antibodies were higher in patients with pulmonary fibrosis and digital ulcers and were associated with disease activity [11,12].

However, it is well known that CXCL4 is a heparin-binding protein [13,14], and heparin-dependent anti-CXCL4 antibodies have been described in another rheumatic disease, systemic lupus erythematosus (SLE) [12].

Most importantly, heparin-dependent antibodies play a pathogenic role in heparin-induced thrombocytopenia (HIT), a rare but fatal autoimmune condition, which leads to massive activation of platelets and thrombi formation with subsequent depletion of platelets [15]. Importantly, the activation of platelets is also characteristic of SSc; thus, whether heparin-dependent antibodies are more frequent in SSc than in the healthy population is of interest [16]. Anti-CXCL4 antibodies that we have previously described in SSc were likely heparin-independent [11,12].

It remains elusive whether antibodies to CXCL4 in SSc can recognize complexes formed by CXCL4 and heparin, what their frequency is, and which role they play in disease pathogenesis. The present research is a pilot study to analyze the occurrence of heparin-dependent autoantibodies in SSc and their possible relationship with heparin-independent antibodies and disease manifestations. We found that heparin-dependent antibodies circulate in SSc and are mainly present in patients with pulmonary arterial hypertension (PAH), and, like the heparin-independent antibodies, correlate with digital ulcers (DU). These observations are of potential interest, as heparin-dependent antibodies are not routinely measured in SSc. We suggest that these antibody types are worth investigating in this chronic condition, characterized by vasculopathy and activation of platelets, in addition to autoimmunity and fibrosis [1,16].

## 2. Materials and Methods

### 2.1. Patients

Blood samples (from 1 to 3 mL) from SSc were from Policlinico Umberto I, Rome, Italy. Plasma or sera from healthy donors (HD), matched for age and sex with SSc as much as possible, were from the “Blood centers”, Policlinico Umberto I, Italy. SSc patients satisfied the American College of Rheumatology (ACR)/European League Against Rheumatism (EULAR) 2013 classification criteria [17]. Plasma was obtained from all of the blood collected in Vacutainer EDTA tubes (Becton and Dickinson, Franklin Lakes, NJ, USA), to avoid clotting. One milliliter of blood was centrifuged at 2000× *g* for 15 min. The supernatant was collected with a pipette and stored in 2 mL tubes at −80 °C for future experiments. Small aliquots of plasma were prepared to avoid freeze-thaw cycles. Exclusion criteria included patients treated with biologics.

### 2.2. IFN-α and TNF-a Determination in Plasma and pDC Cultures

IFN-α was determined by ELISA (MabTech, Cincinnati, OH, USA), as described [5]. TNF-α was measured by using the commercial ELISA by MabTech, (cod. 3425-1H-6, Stockholm, Sweden). Plasma was diluted 1:4 or 1:5 in phosphate buffer solution (PBS) before plating. Culture supernatants were diluted from 1:4 to 1:10 depending on the stimulus used to stimulate pDC.

### 2.3. ELISA for Anti-CXCL4 Autoantibodies and Heparin-Dependent Antibodies in Plasma

Anti-Chemokine (C-X-C motif) ligand 4 (CXCL4) antibodies were measured by an in-house ELISA as described in [11,12]: 96-well flat-bottom plates (non-binding surface polystyrene, Corning, Corning, NY, USA) were coated with 2 µg/mL CXCL4 in carbonate buffer (0.1 M NaHCHO_3_, pH 9) for 2 h (or overnight) and washed four times with PBS + 0.1% Tween-20. CXCL4 was synthesized by Biomatik. Blocking buffer containing 2% bovine serum albumin (BSA, Sigma-Aldrich, St. Louis, MO, USA) in PBS was used for at least 1 h (or overnight) to saturate unspecific binding sites. After washing, sera or plasma were diluted 1:100 in PBS + 2% BSA, followed by a 1 h incubation with a horseradish peroxidase (HRP)-conjugated goat anti-human IgG (Sigma-Aldrich, St. Louis, MO, USA), diluted 1:5000 in PBS. The color was developed for 5 min with 3,3′,5,5′-tetramethylbenzidine (TMB) substrate (Sigma-Aldrich, St. Louis, MO, USA). The reaction was stopped by adding 50 µL of 2 N H_2_SO_4_. Absorbance was determined at 450 nm with a reference wavelength of 540 nm. Anti-CXCL4 antibodies were considered positive when OD exceeded the mean OD values obtained with HD plasma, plus two standard deviations (SD).

Heparin-dependent anti-CXCL4 antibodies were determined by using a commercial kit from MyBioSource (Human Heparin-PF4 Complex: HIT antibody ELISA Kit, San Diego, CA, USA).

Significant presence of these antibodies in SSc was calculated with respect to the mean of expression in HD plasma

### 2.4. Isolation of pDCs and Their Stimulation

For isolation of human peripheral blood pDC, blood buffy coats of healthy donors (HD) were obtained from Blood Center of Policlinico Umberto I, Rome, IT. After separation of PBMCs by Ficoll centrifugation, pDC were purified as described [5], by using a Diamond Plasmacytoid Dendritic Cell Isolation Kit (Miltenyi Biotec, Bergisch Gladbach, Germany). Purified pDC were seeded into 96-well round-bottom plates with 250 × 10^3^ cells per ml. CXCL4 was premixed with total human DNA (10 µg per mL) and added to the pDC cultures after 15-min incubation at room temperature [5]. The concentration of CXCL4 was between 1–2 µM. Complexes were also formed with low molecular weight heparin (calcium nadroparin, 1-5-25-125 UI/mL), as described [11]. Moreover, cells were treated with CXCL4–DNA complexes, in the presence or absence of heparin. Cells were also stimulated with control CPGA (from Invivogen), a TLR9 artificial stimulator.

### 2.5. Measurement of CXCL4 in Plasma of SSc Patients

CXCL4 in plasma was tested by diluting plasma 1:100 in PBS, using the Human CXCL4 DuoSet Elisa, R&D Systems (Minneapolis, MN, USA) [5].

### 2.6. Statistical Analyses

Differences between mean values were assessed by Wilcoxon’s matched-pairs signed rank test, or Mann–Whitney test (one tailed or two tailed), especially in cases of low patient sample sizes. Statistical significance was set at *p* ≤ 0.05. Correlation analyses were performed by Pearson’s or Spearman’s rank correlation tests, depending on the sample size (N). For low sample sizes, we always used Spearman’s correlation. Data were analyzed and correlations were performed using GraphPad Prism 7.0. For differences of distribution of antibody reactivity in different subgroups of patients we used the *Chi-square* test (one tailed). 

## 3. Results

### 3.1. Plasma Heparin-Dependent Antibodies Are Present in SSc Patients

We have analyzed the presence of heparin-dependent anti-CXCL4 antibodies using a commercial ELISA test (see Methods), in a cohort of 33 SSc patients and 27 controls HD (see Table 1 for the characteristics of the SSc-patients cohort). 

**Figure 1:**
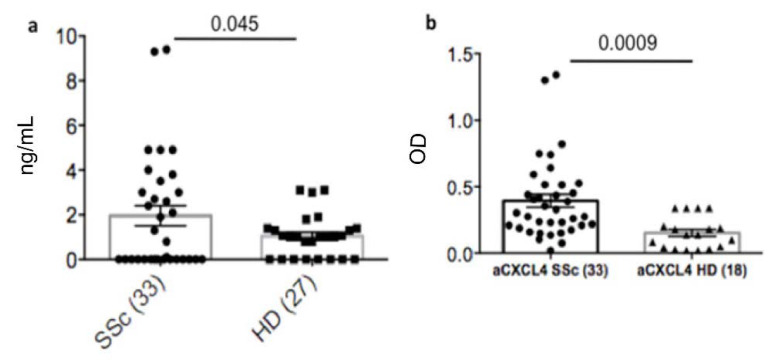
Heparin-dependent anti-CXCL4 antibodies are present in SSc patients. Plasma of SSc patients and HD were tested for the presence of heparin-dependent (**a**) and heparin-independent (**b**) antibodies by using a commercial (**a**) or an in-house (**b**) ELISA [7,8]. Horizontal bars are the means, vertical bars are the standard error of the mean (SEM), *p* values (indicated in the graphs) were calculated by Student’s *t*-test for unpaired samples (two tails).

The results in Figure 1a show that although HD can express these antibodies, SSc patients have a significantly higher concentration of heparin-dependent anti-CXCL4 antibodies (also referred to HIT antibodies) as compared to the HD counterpart. In particular, by using as cut-off the expression level of heparin-dependent antibodies in HD (plus two standard-deviations, SD, see Methods), we found that 8 out of 33 (24%) SSc patients had a higher concentration of these antibodies. Considering only these positive responses in both groups (SSc patients and HD), a more significant difference between the two groups was evident (Mann-Whitney test: *p* = 0.0003). 

In parallel, (Figure 1b) we have checked the presence of what we call heparin-independent anti-CXCL4 antibodies in the same SSc patients by using our previously published in house assay [4,5]. Ten out of 33 SSc-patients (30%) responded to CXCL4 alone, as compared to HD. Even in this case, positivity was calculated by using HD as a reference. SSc-patients were considered positive for anti-CXCL4 antibodies when the optical density (OD) was above the value of the mean of the OD measured in HD plus two standard deviations (as described [11,12]).

These results confirm that CXCL4 is a B-cell autoantigen in SSc and show that antibodies to CXCL4 can be either heparin-dependent or heparin-independent in SSc. 

### 3.2. Plasma Heparin-Dependent Antibodies Inversely Correlate with Heparin-Independent Antibodies to CXCL4

**Figure 2:**
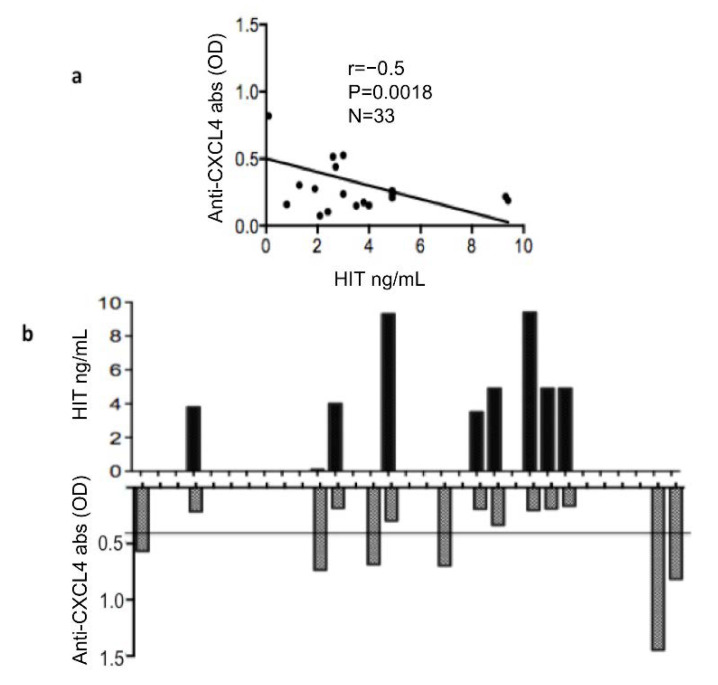
Plasma heparin-dependent antibodies inversely correlate with heparin-independent antibodies to CXCL4. OD values of heparin-independent antibodies (anti-CXCL4 abs OD) were plotted against values of heparin-dependent abs (HIT abs) and Spearman’s correlation test was used to address correlation between these antibody types (**a**). The “r” coefficient of correlation, *p* (significance) and N (sample size) are reported on the graph. (**b**) Histograms showing the positivity for HIT antibodies and anti-CXCL4 antibodies to show that positivity for anti-CXCL4 and for HIT antibodies are mutually exclusive.

**Figure 3:**
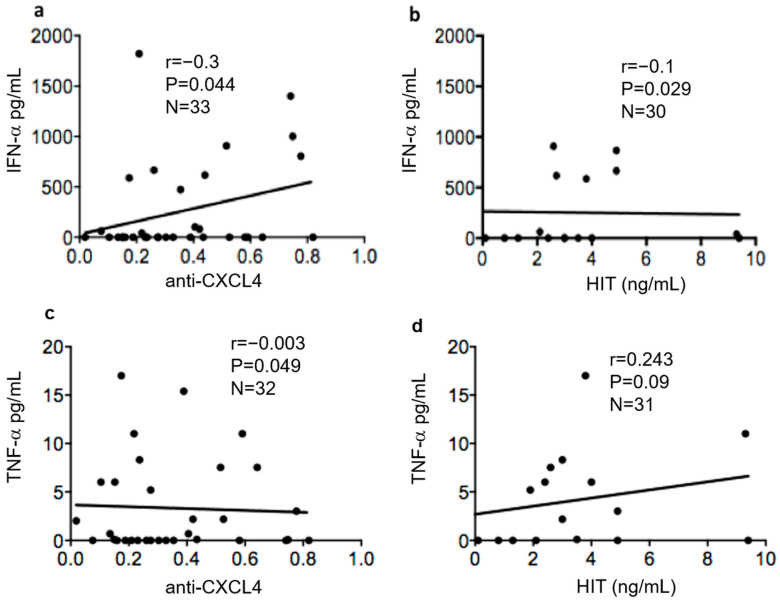
HIT antibodies do not correlate with IFN-α in plasma. Plasma of SSc was assessed for the positivity for IFN-α and TNF-α by ELISA (see methods). Values of IFN-α and TNF-α concentrations were plotted against values of anti-CXCL4 positivity (**a**,**c**) or amounts of HIT antibodies (**b**,**d**). Spearman’s correlation test was used to address correlation between these antibody types and IFN-α. The “r” coefficient of correlation, *p* (significance) and N (sample size) are reported on each graph.

We than assessed whether there was a correlation between heparin-dependent and heparin-independent (HIT) antibodies to CXCL4. Interestingly, the correlation between these two antibody types was inverse and significant (Figure 2a). In addition, when considering only the people positive for heparin-dependent antibodies, the inverse correlation was stronger (r = −0.75, *p* = 0.001, *n* = 8). Indeed, this reflects the fact that none of the heparin-dependent positive patients expressed heparin-independent antibodies (as depicted in Figure 2b). This result suggests that heparin-dependent and heparin-independent anti-CXCL4 antibodies are mutually exclusive, at least in the cohort analyzed. Next, we assessed whether the two antibody types correlated with IFN-α measured by ELISA in the same SSc plasma. We found a certain correlation between anti-CXCL4 antibodies and IFN-α, as previously described, but correlation was completely absent when we considered the heparin-dependent anti-CXCL4 antibodies (Figure 3a,b, respectively). These results can suggest that these two types of anti-CXCL4 antibodies may have different effects, and are likely generated by different mechanisms. To avoid bias due to cytokine measurement, we also measured plasma concentrations of TNF-α. This cytokine is usually upregulated in SSc and plays a role in the pathogenesis [18]. When we performed a correlation between presence of anti-CXCL4, or heparin-dependent anti-CXCL4 antibodies (HIT) and TNF-α concentrations, we did not find any correlation (Figure 3c,d). This suggests that the correlation between anti-CXCL4 antibodies that are heparin-independent and IFN-α is a constant phenomenon in SSc, which is not present for other cytokines. IL-6 was measured in this cohort and was very low, so we did not calculate this correlation with IL-6.

### 3.3. Heparin Blocks the Stimulatory Ability of CXCL4-DNA Complexes on IFN-α Production by pDCs

**Figure 4:**
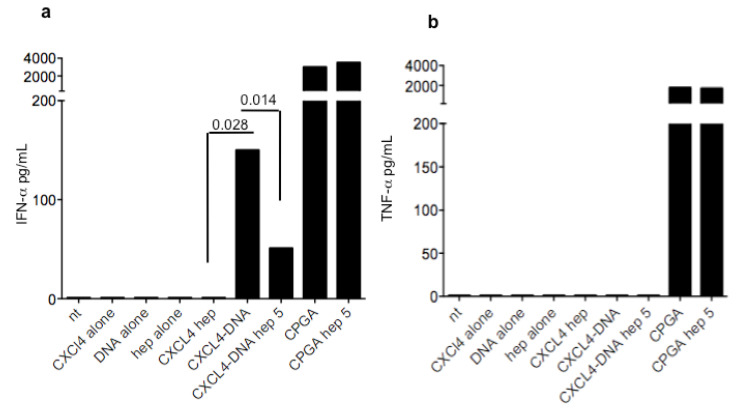
CXCL4-heparin complexes do not stimulate pDC and heparin blocks stimulation by CXCL4-DNA complexes. PDC were cultured with the indicated stimuli and both IFN-α (**a**) and TNF-α (**b**) were assessed on culture supernatants 24 h later by ELISA (see methods). Values of IFN-α and TNF-α concentrations are indicated. Heparin was used at 5 U/mL. Histograms are representative of four independent experiments performed with pDC from different healthy donors. Significance (calculate by Mann-Whitney test) is reported on the graph.

We have previously demonstrated that CXCL4-DNA complexes do activate pDC to produce IFN-α [5]. We have also demonstrated that an antibody for CXCL4 increases the stimulatory activity of CXCL4-DNA complexes, presumably because of a better uptake of these complexes [11]. We reasoned that HIT antibodies, which exclusively depend on heparin for binding to CXCL4, would preferentially mediate the uptake of heparin into the cells. Thus, the reason why they do not correlate with IFN-α could be due to a possible inhibitory activity of heparin on CXCL4-DNA complexes’ interferogenic activity. Here, we show that not only do CXCL4-heparin complexes not stimulate pDC, but that heparin effectively competes with DNA for binding to CXCL4 (Figure 4), as an addition of 5 U/mL of heparin significantly reduced the IFN-α production by pDCs. This effect was negligible for the DNA mimic CPGA, which triggers TLR9 independently from binding to CXCL4. Thus, antibodies for CXCL4-heparin complexes may favor heparin uptake into the cells, which may block the activity of internalized CXCL4-DNA complexes. 

### 3.4. Heparin-Dependent (HIT) Antibodies Correlate with Digital Ulcers

**Figure 5:**
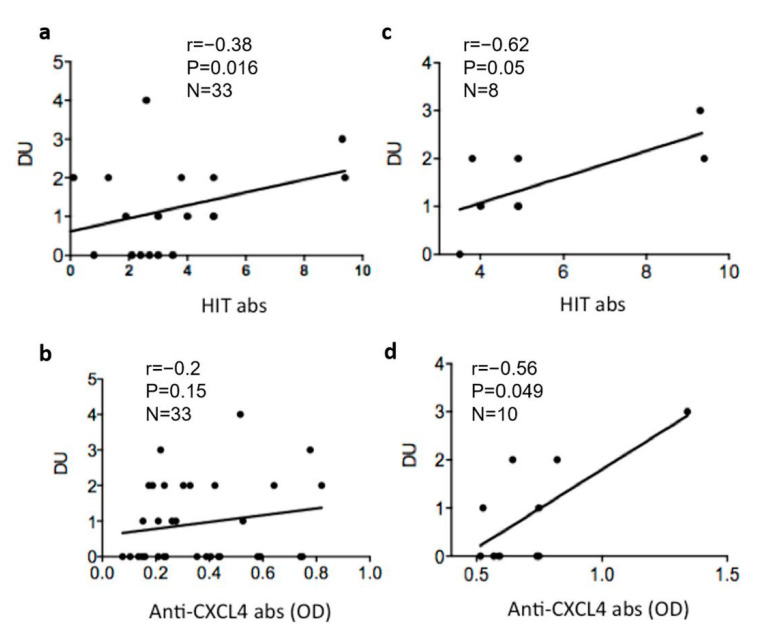
Heparin-dependent (HIT) antibodies correlate with digital ulcers. Numbers of digital ulcers (DU) was plotted against values of HIT (**a**,**c**) antibodies or anti-CXCL4 (**b**,**d**) antibody response (expressed as OD). Spearman’s correlation test was used to address correlation between these antibody types and DU. The “r” coefficient of correlation, *p* (significance) and N (sample size) are reported on the graphs. In the right-hand graphs only patients with positivity for the antibodies are reported.

Next, we tested whether heparin-dependent anti-CXCL4 (HIT) antibodies correlated with number of DU. A moderate and significant correlation was observed (Figure 5a). This correlation was more consistent when only the positive SSc-patients were selected (Figure 5b). These results suggest that phenomena mediated by heparin-dependent anti-CXCL4 antibodies could be important in determining the formation of ulcers. We have previously shown that anti-CXCL4 antibodies (independent from heparin) were higher in patients with DU. Here, we addressed whether in this cohort the heparin-independent anti-CXCL4 antibodies also correlated with the number of DU. Although the correlation was not observed considering the entire cohort (Figure 5c), when we considered only the anti-CXCL4 positive patients, we found a certain correlation, which appeared significant (Figure 5d).

**Figure 6:**
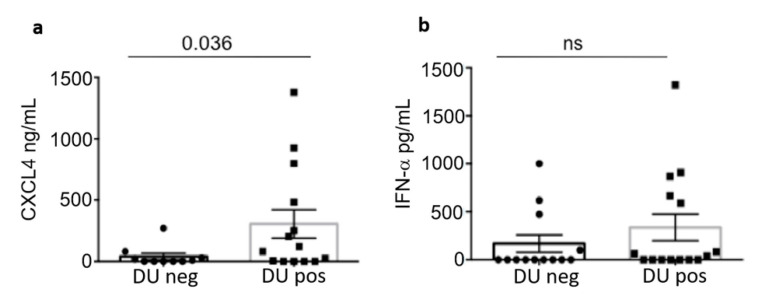
The autoantigen CXCL4 is more expressed in patients with DU. CXCL4 was measured by ELISA in patients with DU (DU pos) or without DU (DU neg) (**a**). Differences between the two groups was assessed by Mann-Whitney’s test. Significance *p* is reported, ns (non-significant). (**b**) Differences in IFN-α levels, measured by ELISA as above, between the two groups.

Of note (Figure 6a) is that patients with DU were also those expressing the most elevated CXCL4 concentration in plasma; IFN-α tended also to be higher in SSc patients with DU, as compared to patients without DU, but the difference observed was not significant (Figure 5b). These findings seem to suggest that the formation of DU is likely to be linked to CXCL4 expression and functions, but also depends on the type of the antibody response to CXCL4.

### 3.5. Heparin-Dependent Anti-CXCL4 (HIT) Antibodies Are Linked to Pulmonary Artherial Hypertension (PAH)

**Figure 7:**
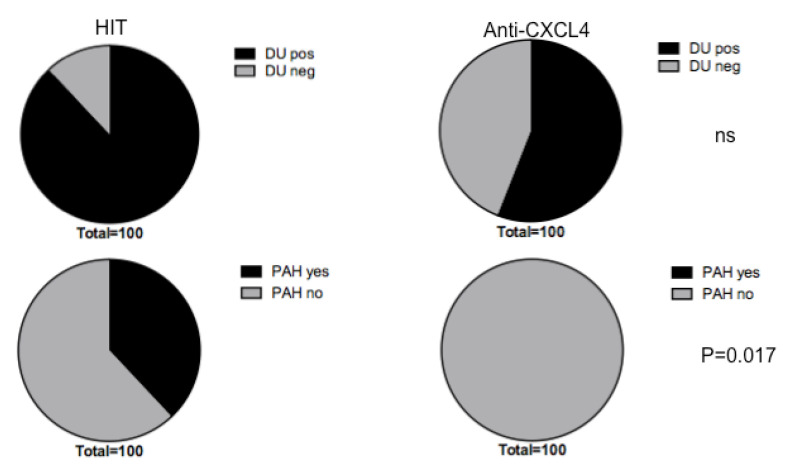
Heparin-dependent anti-CXCL4 antibodies may be linked to PAH. Percent of patients expressing HIT or heparin-independent anti-CXCL4 antibodies (anti-CXCL4) is shown in DU positive and negative patients, upper pie diagrams, or in patients with PAH or with no PAH, lower pie diagrams. More than 1/3 of PAH positive patients have HIT antibodies and the difference, calculated by Chi-square’s test is significant. *p* values reported in the figure.

We have demonstrated in the previous paragraph that anti-CXCL4 antibodies, especially the HIT, correlated with DU in SSc. Considering the DU-positive SSc-population, as shown in the pie diagram in Figure 6a, we found that heparin-dependent anti-CXCL4 antibodies (HIT) were present in the majority of DU-positive patients (88%). DU positive patients also expressed anti-CXCL4 antibodies that are heparin-independent in 56% of cases. A higher expression by SSc patients with DU of HIT antibodies, as compared to heparin-independent anti-CXCL4 antibodies, is therefore in keeping with the data of correlation of the previous paragraph. However, although there is a difference in the frequency of the two antibody types with respect to the presence of DU, this difference, calculated with a Chi-square’s test, was not significant. We looked at other disease parameters and we found no significant differences either. A significant difference become apparent when we looked at PAH. We found that 38% of the patients with PAH presented heparin-dependent antibodies (HIT) (black in the pie diagram in Figure 7, lower panel diagram). Patients with PAH did not have significant levels of heparin-independent anti-CXCL4 antibodies. The difference between the two situations, namely the exclusive presence of HIT antibodies in patients with PAH, was found to be significant by Chi-square’s test. These results could suggest that HIT antibodies can have a function in PAH, or could be potential markers of this condition.

## 4. Discussion

In this pilot study, by analyzing a SSc cohort, we have examined the expression of heparin-dependent anti-CXCL4 antibodies, also known as HIT antibodies, and their relationship with disease in SSc patients [13]. CXCL4 is a marker of SSc, which especially predicts SSc progression and complications [3]. In our previous studies [11,12], we only assessed the presence of antibodies directed to CXCL4, but not to CXCL4 and heparin. However, heparin is a CXCL4 binding protein, and anti-CXCL4 heparin-dependent antibodies could be also generated in SSc. They have been found in SLE patients and are characteristic of the rare autoimmune disease called heparin-induced thrombocytopenia (HIT) [13,14].

SSc patients that do not have significant amounts of anti-CXCL4 antibodies usually have HIT antibodies, at least in our cohort. If this is a general phenomenon, it needs to be studied in larger cohorts. This evidence may mean that the two antibody types are probably generated via different mechanisms. We hypothesize that generation of CXCL4-specific antibodies, which are heparin independent, can be due to formation of complexes between CXCL4 and nucleic acids, as in SSc there is a high expression of CXCL4 and circulating and tissue-deposited CXCL4-DNA complexes [5]. This may favor breach of tolerance to CXCL4. It is likely that, in a very inflamed context and in the presence of cell death, CXCL4 easily binds nucleic acids released in high quantities by dead cells. Antibodies raised may transport self-DNA inside the immune cells (pDC, B-cells). This has been suggested in our previous paper, where the addition of an anti-CXCL4 antibody to pDC treated with CXCL4-DNA complexes implemented pDC activation [11]. Whether HIT antibodies cross-recognize anti-CXCL4-DNA complexes is unclear for the moment. However, if the HIT bind primarily CXCL4-heparin complexes, it is likely that they will favor uptake of heparin in pDC and other immune cells. Here we show not only that CXCL4-heparin complexes do not stimulate pDC, but also that heparin blocks the interferogenic activity of CXCL4-DNA complexes. This can give an explanation of why only heparin-independent anti-CXCL4 antibodies correlate with IFN-I. That heparin can antagonize the TLR9 stimulatory ability of CXCL4 and DNA had been already observed with B-cells in our previous paper [11]. 

We concentrated our attention on the capacity of both CXCL4-DNA and anti-CXCL4 antibodies to up-regulate IFN-α because immune complexes formed by CXCL4-DNA or CXCL4-DNA-antibody are likely to render self-DNA immunogenic and favor activation of pDC. We do not expect these immune complexes to stimulate (at least in pDC) other cytokines such as, for instance, TNF-α. Indeed, human DNA fails to induce significant production of TNF-α by pDC [18], even when in a complex with specific antimicrobial peptides that also bind, like CXCL4, the DNA, protecting it from degradation. This is also true for CXCL4, as shown here, where the complex with DNA of human origin only stimulate IFN-α but not TNF-α unlike artificial DNA mimics, such as CpG molecules, in our experiment CPGA. Indeed, we found no correlation between anti-CXCL4 antibodies and TNF-α, a cytokine also involved in SSc [19], and expressed in plasma of SSc patients, which reinforces the role of CXCL4 itself and anti-CXCL4 antibodies in participating, in particular, to sustain an IFN-I signature [11,12].

On the other hand, we already published that CXCL4-DNA, but not CXCL4-heparin complexes, can directly stimulate memory B-cells to become antibody-secreting plasma cells [11,12]. The production of IFN-α, which can be at least partially ascribed to the presence of CXCL4-DNA (and perhaps CXCL4-RNA) complexes in SSc, can increase the capacity of B-cells to produce autoantibodies [11]. Thus, the relationship between anti-CXCL4 antibodies and IFN-α can be explained in an additional way: the creation of a loop in which CXCL4-DNA complexes stimulate pDC and IFN-α, the IFN-α stimulates B-cells to produce more antibodies to CXCL4, and the latter, in turn, further amplify IFN-α production.

This picture reinforces the role of IFN-I in SSc, a cytokine that when present at disease onset is prognostic of a more severe disease at later stages [7,8,9].

Regarding the apparent reciprocal expression of CXCL4 and HIT antibodies, it is interesting to report a mechanism that could also explain this mutually exclusive expression. Sachais et al. [20] showed that some HIT antibodies also bind to CXCL4 alone, but with much lower affinity than to CXCL4-heparin complexes. Antibodies with such a behavior were shown to induce clustering of CXCL4. In other words, they acted like heparin, inducing CXCL4 oligomerization, which increases immunogenicity. This action promotes the generation of further epitopes by cross-linking CXCL4 tetramers. Given the high likelihood of generating anti-CXCL4 antibodies in SSc, as CXCL4 is over-expressed, this mechanism could be frequently operative in SSc and favor the production of HIT antibodies.

Both antibody types were instead found to correlate with DU. In this paper and in previous studies, we have shown that anti-CXCL4 antibodies correlated with DU [11,12]. We reasoned that this could be due to the fact that anti-CXCL4 antibodies favor IFN-α production, in that DU-positive patients usually show a higher IFN-α signature. However, HIT antibodies also seem to be frequent in DU-positive patients. In this case, the role of IFN-α could be important, but so could the presence of HIT. Two different mechanisms could promote DU: one linked to IFN-α, or at least to more general TLR-stimulation induced by CXCL4 itself and CXCL4-directed antibodies, and one mediated by the HIT antibodies, which are more linked to platelets’ activation and thrombi formation [13]. It is interesting that HIT antibodies were especially present in patients with PAH, which may likely be linked to the intrinsic pro-thrombotic effect mediated by the HIT antibodies.

The limitations of this study are the small cohort studied and the fact that HIT antibodies should also be studied for their capacity to activate platelets in functional assays [21,22].

Nevertheless, we believe that HIT antibodies, which are increased in SSc as compared to HD, deserve more attention with respect to their clinical relevance as markers of specific disease features, and to prevent complications in SSc patients due to massive platelet activation.

## Figures and Tables

**Table 1:** Selected main clinical, demographic and laboratory features of SSc patients and HD.

Clinical and Demographic Characteristics of Patients and Controls ^1^	SSc (33)	HD (27)
Age, mean (range): years	50 (32–61)	40 (29–55)
Sex (M/F):	0/33	3/24
SSc Form (limited/diffuse)	1/32	N/A
DU	55%	N/A
Lung fibrosis (%)	33%	N/A
PAH	25%	N/A
ATA	70%	N/A
ACA	5%	N/A
aRNAP3 positivity	2%	N/A
Raynaud’s phenomenon	94%	N/A
DMARDs	99%	N/A

^1^ Legend: Ea SSc, early diffuse SSc; ACA, anti-centromere antibodies; ATA, anti-topoisomerase antibodies; anti-RNAP3, anti-RNA-polymerase 3; DU, digital ulcers; DMARDS, disease-modifying anti-rheumatic drugs; PAH, pulmonary arterial hypertension; N/A, not applicable.

## Data Availability

Data of ELISA can be provided upon reasonable request.
